# Cost-effectiveness of rotavirus vaccination in the Netherlands; the results of a consensus model

**DOI:** 10.1186/1471-2458-11-462

**Published:** 2011-06-10

**Authors:** Mark H Rozenbaum, Marie-Josee J Mangen, Carlo Giaquinto, Jan C Wilschut, Eelko Hak, Maarten J Postma

**Affiliations:** 1 Unit of PharmacoEpidemiology & PharmacoEconomics (PE2), Department of Pharmacy, University of Groningen, Groningen, The Netherlands; 2 University Medical Center Utrecht, Julius Center for Health Sciences and Primary Care, Utrecht, The Netherlands; 3 Department of Paediatrics, Università degli Studi di Padova, Padova, Italy; 4 Department of Medical Microbiology, Molecular Virology Section, University Medical Center Groningen, University of Groningen, Groningen, The Netherlands; 5 CoRoVa = Consensus on Rotavirus Vaccination

## Abstract

**Background:**

Each year rotavirus gastroenteritis results in thousands of paediatric hospitalisations and primary care visits in the Netherlands. While two vaccines against rotavirus are registered, routine immunisation of infants has not yet been implemented. Existing cost-effectiveness studies showed inconsistent results for these vaccines because of lack of consensus on the impact. We aimed to investigate which factors had a major impact on cost-effectiveness and were primarily responsible for the large differences in previously estimated cost-effectiveness ratios.

**Methods:**

Based on updated data on health outcomes and cost estimates, we re-assessed the cost-effectiveness of routine paediatric rotavirus vaccination within the National Immunization Program for the Netherlands. Two consensus meetings were organised with national and international experts in the field to achieve consensus and resolve potential controversies.

**Results:**

It was estimated that rotavirus vaccination in the Netherlands could avert 34,214 cases of rotavirus gastroenteritis in children aged less than 5 years. Notably, 2,779 hospitalisations were averted of which 315 were extensions of existing hospital stays due to nosocomial rotavirus infection. With a threshold varying from 20K€ - 50K€ per QALY and according to the base-case scenario, the full vaccination costs per child leading to cost-effectiveness was €57.76 -€77.71. Results were sensitive to the inclusion of potential vaccine induced herd protection, QALY losses and number of deaths associated with rotavirus gastroenteritis.

**Conclusions:**

Our economic analysis indicates that inclusion of rotavirus vaccination in the Dutch National Immunization Program might be cost-effective depending on the cost of the vaccine and the impact of rotavirus gastroenteritis on children's quality of life.

## Background

In 2008, approximately 8.8 million children died before reaching their fifth birthday worldwide [1]. After pneumonia, diarrhoea is the second leading cause of mortality in these children with approximately 1.4 million deaths annually of which approximately 500,000 are due to rotavirus (RV) infection [1-3]. While in Western countries mortality due to diarrhoea is low, a high level of morbidity has led scientific societies (ESPID and ESPGHAN) to recommend the introduction of universal mass vaccination with rotavirus vaccines to all Western European infants and children [4,5]. One of the factors influencing the decision to introduce a new vaccine for infants, such as the RV vaccine, into the Dutch National Immunization Program (NIP) involves an acceptable cost-effectiveness profile under current standards [6].

Over the last few years, four different studies were performed to assess the cost-effectiveness of routine infant RV vaccination in the Netherlands, and reported inconsistent and varying results [7-10]. For example, Goossens *et al. *concluded that mass vaccination against rotavirus gastroenteritis (RVGE) can be attractive from both an economic and a health care point of view, while a more recent paper by Mangen *et al. *stated that vaccination cannot be considered cost-effective [7,9]. Though the four studies focused on either one or both of the two registered vaccines (RotaTeq^®^, Merck & Co, Inc, Whitehouse Station, NJ; and Rotarix^®^, GlaxoSmithKline Biologicals; Rixensart, Belgium), differences in cost-effectiveness between the vaccines appeared small and resulted, next to the used vaccination schedules and genotype-specific efficacy, mainly from assumed between-dose efficacy estimates. Very recently, new data from additional trial analyses showed even smaller differences in efficacy between both individual vaccines [11]. We therefore updated the cost-effectiveness analyses of RV vaccination for the Netherlands assuming absence of differences between the two vaccines. Two consensus meetings were held with national and international experts in the field, from academia, clinical backgrounds, industry and health policy groups to reach consensus on final assumptions and resolve any remaining controversies. Explicitly, we investigated the most important factors in the analyses and those parameters primarily responsible for the large differences between the cost-effectiveness estimates of the various models.

## Methods

### Model

**Figure 1:**
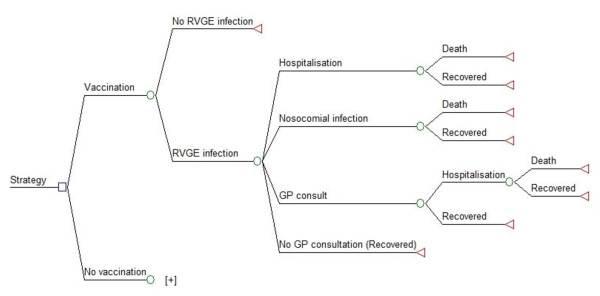
**Schematic overview of the model**. The boxes represent nodes, with blue squares indicating decision nodes, with green circles indicating probabilistic nodes and red triangles indicating end nodes. The "No vaccination" arm is a clone of the "Vaccination" arm (as represented by the + sign).

An age-structured cohort model was developed in Excel for a hypothetical cohort of 180,000 newborns, which approximates the annual Dutch birth cohort (Figure 1). A birth cohort is included in the model and two strategies were compared: one being the current situation without vaccination (current situation), and the other being mass universal RV vaccination within the framework of the Dutch NIP. The time horizon of the model was 5 years with time cycles of 1 month for children less than 1 year of age and annual analysis thereafter. Outcomes in our analysis were classified by severity and included home-treated community-acquired diarrhoea and RV infection leading to consultation of a general practitioner (GP) and/or hospital admissions (including emergency department [ED] visits), nosocomial infections and death. Multiple outcomes per RV infection are possible in the model, such as the number of GP visits and hospitalisations.

### Epidemiology

Four recent Dutch cost-effectiveness analyses [7-10] showed a large variation in the incidence of RV despite the fact that the investigators based their epidemiological estimates on similar sources [12-15]. In particular, the incidence of community-acquired infections resulting in a GP visit differed considerably, which was related to different assumptions regarding underreporting and extrapolation of the relatively outdated incidence data to the present. Due to the absence of more recent data, we chose to include the conservative incidence numbers based on the recent analysis by Mangen *et al. *[9]. These authors made their epidemiological estimations on the basis of a re-analysis of raw data from previous epidemiological studies [13,14]. In Table 1, the incidence of specific categories of RVGE-cases (*e.g. *number of cases treated at home, GP visits, and hospitalisations) is shown for children aged less than 5 years.

**Table 1:** Parameters used in the economic model

Description	Base case value	Distribution	References
Vaccine Efficacy			

Severe infections hospitalisation (first year)	0.945	Lognormal mean 0.945 (SE 0.014)	[21]

Waning rate per year (exponential decrease)	0	NA	[23]

Mild infections requiring an office visit (average first 2 years, see Methods)	0.874	Lognormal mean 0.874 (SE 0.052)	[24]

Waning rate per year (exponential decrease)	0.09	NA	Assumption

Mild infections treated at home (first year)	0.720	Lognormal mean 0.720 (SE 0.040)	[21]

Waning rate per year (exponential decrease)	0.18	NA	[21]

			

Incidence per million children (<5 years)			

Total number of community-acquired RV cases	65,680	Normalised mean: 65,680 (90%CI; 43,890-90,945)^a^	[9]

No medical help requested	52,947	Total number of cases minus total number of GP visits (calculated)	

GP visits	12,733	Normalised mean: 12,733 (90%CI; 6,922-20,384)^a^	[9]

Total hospitalisations	3600	Pert (2600; 3600; 4500)	[9]

Of which nosocomial infections	13%	NA	[16]

Deaths as% of total number of hospitalisations	0.02%	Triangular (0%; 0.02%; 0.12%)	[18]

			

Total QALY detriment			

Rotavirus infection treated at home			[25,26]
0-18 months	0.0015	See Methods section	
18-59 months	0.0025		

Rotavirus infection requiring medical attention (GP)			[25,26]
0-18 months	0.0022		
18-59 months	0.0031	See Methods section	

Rotavirus infection requiring hospitalisation (including nosocomial)			[25,26]
0-18 months	0.0036	See Methods section	
18-59 months	0.0042		

Total direct costs per case			

Case treated at home (0-3 years)^b^	4.25	Triangular (2.66; 4.25; 7.44)	[7]

Case requiring GP visit^b^	70.08	Triangular (52.08; 70.08; 82.70)	[7,28]

Case requiring hospitalisation	2146	Triangular (1933; 2146; 2359)	[7,16,42]

Case requiring hospitalisation (nosocomial)	1825	Triangular (1280; 1825; 2377)	[7]

Total indirect cost per case (care giver taking care of child)			

Case treated at home	35.26	Triangular (31.74; 35.26; 38.79)	[28,42]

Case requiring GP visit	51.09	Triangular (45.99; 51.09; 59.20)	[28,42]

Case requiring hospitalisation^c^	55.41	Triangular (49.87; 55.41; 60.95)	[28,42]

Case requiring hospitalisation (nosocomial)	45.34	Triangular (40.80; 45.34; 49.87)	[28,42]

Total cost per vaccinee	50, 75, 100	Triangular (50; 75; 100)	Assumption

NA, not applicable; SE, standard error

^a ^Square root transformation was applied.

^b ^Cost for older children were lower as diapers were not assumed to be used any more in children aged 3 years and older.

^c ^In the model indirect costs and QALYs are corrected for indirect costs and QALYs which have already occurred at the GP to avoid double counting (as we assumed that all hospitalised cases would already have visited a GP before being hospitalised).

In order to calculate age-specific disease distributions we applied the age-specific hospitalisation distribution which divided the total estimated number of cases by the different age groups [16]. It was therefore implicitly assumed that this distribution would be comparable for hospitalisations, GP visits and cases treated at home. As the distribution for the nosocomial infections substantially differs from community-acquired RV infection [17], the age-distribution for nosocomial infections was based on specific Dutch nosocomial admission data [16]. We assumed that fatal infections would only occur in hospitalised children. As specific Dutch mortality data is lacking, we applied a mortality rate of 0.02% for hospitalised children based on the hospitalised mortality rate due to RV infection (as primary diagnosis) observed in England and Wales [18]. Similar estimates were recently found for other Western European countries [19,20]. These estimates are all much lower than those used in the previous Dutch cost-effectiveness studies [7-10].

### Vaccine efficacy, waning immunity and between-dose efficacy

We matched the specific types of disease cases with the most appropriate vaccine efficacy estimates, preferably based on clinical trial data gathered in European countries rather than from other continents. For cases resulting in a GP visit or a hospitalisation, efficacy was based on the observed reductions in health care use in the trials, while efficacy against cases treated at home was based on that shown against RVGE cases of any severity.

The vaccine efficacy against RVGE cases treated at home was recently estimated to be 72.0% (95% confidence interval [CI] 63.2%-78.9%) during the first full season after vaccination and 58.5% (95%CI 40.1%-74.4%) during the second full season [21]. Based on the difference in efficacy between first and second RV seasons after vaccination, we conservatively assumed that the vaccine efficacy would exponentially decrease by 18.8% per year starting after the first year [21].

Against hospitalisation we applied an efficacy of 94.5% (95%CI: 91.3%-96.8%) based on the rate reduction in hospitalisations and ED visits observed in European children [21]. Based on data for the first 3 years, we assumed that this efficacy would remain stable during the first 5 years and thus no waning immunity was assumed [22]. This assumption is further supported by a recent study which shows that the efficacy against hospitalisations and ED visits was similar in the first and second year after vaccination [23]. Efficacy against cases requiring a GP visit was shown to be 87.4% (95%CI: 75.5%-95.7%) up to 2 years after vaccination [24]. As no specific waning data are available for this case definition, we assumed that the waning rate would be 9.4%, which is the mid-point of the more severe cases (hospitalised) and mild cases (treated at home). To be consistent, we increased the first-year efficacy to 90.7% so that the average vaccine efficacy over 2 years would be equal to 87.4%. We note that in reality, waning might be much lower (see below), therefore the impact of reducing the waning rate was also explored.

Previous analyses on cost-effectiveness assumed slightly lower between-dose efficacies for RotaTeq^® ^compared to Rotarix^®^, resulting in a more unfavourable cost-effectiveness estimate for RotaTeq^® ^[8,9]. However, recent data suggest that between-dose efficacies for infections resulting in ED and hospitalisation visits is much higher for RotaTeq^® ^than was previously assumed [11]. The efficacy of RotaTeq^® ^against hospitalisations and/or ED visits between dose 1 and dose 2 was estimated to be 82% (95%CI: 39-97%), and 88% (95%CI: 68-96%) between dose 2 and dose 3 [11]. This corresponds to an efficacy proportion after the first dose of 86% (82/95*100%) and 92% (88/95*100%) between the second and the third dose. We also applied these proportions for the between-dose efficacies for infection requiring a GP visit and cases treated at home.

A vaccine uptake rate of 95% was applied, which means that in our model, 95% of all children receive all doses and 5% do not get any doses. The 95% of children receiving all doses were assumed to be vaccinated at 2, 3 and 4 months.

### QALY losses

Two studies estimating health-related quality-of-life losses in children suffering from RVGE have been performed [25,26]. A recent study in the UK estimated the quality of life in young children up to the age of 5 with RVGE using the EQ-5D [26], with 25 GPs as proxies. The study differentiated according to disease severity (primary care request only or hospitalisation) and age (0-18 months; 18 months to 5 years). Goossens *et al. *used these estimates in their cost-effectiveness analysis of RV vaccination [7]. However, most of the RV cost-effectiveness analysis studies used the quality-adjusted life year (QALY) weights derived by Brisson *et al. *[25]. In this Canadian study, caregivers evaluated health-related quality of life in their children and themselves. Children (<36 months) and caregivers were included from 59 participating practices (both family physicians and paediatricians) when presenting with RVGE [25]. The study estimated the QALY loss in children suffering from RVGE at 0.0022. No differentiation between disease severity or age was made. As no data are available, most previous health economic studies used this QALY decrease for cases needing medical attention (both GP and hospitalisation), and reduced it by 50% for cases requiring no medical help. This approach might be too conservative for hospitalised cases, since case inclusion took place when visiting primary care (although some cases might also have been referred to a hospital). Furthermore, in case of the Netherlands, the QALY decrement previously used for cases treated at home is likely to be too conservative since only the most severe RVGE cases are expected to visit a primary care facility in the Netherlands (possibly related to the fact that no medical certificate is required for staying at home to care for a sick child and thus caregivers do not feel the need to visit the GP except for the most severe cases).

To be conservative, we based our QALY estimates on the Canadian study, but applied a correction factor for age and severity (hospitalised cases) on the basis of the UK study (see Table 1 for specific QALY decrements). For cases requiring no medical help, we assumed that the QALY loss would be 31% lower than for cases requiring a GP visit. This was based on the relative duration of illness for cases visiting a GP being 7.1 days and cases treated at home being 4.9 days [9,27]. We note however that this estimate is still likely to be too conservative, especially as in the Netherlands.

We did not include QALY decrements for caregivers in our base-case analysis. However, we did explore the impact on the incremental cost-effectiveness ratio (ICER) of including these estimates in a scenario analysis [25]. We also investigated the impact of including the QALY decrements based on either the Canadian or the UK estimates, and the impact of including non-age-or sex weighted disability-adjusted life years (DALYs) rather than QALYs based on the study by Mangen *et al. *[9]. To be conservative and fully in line with the design of the studies estimating QALY, we only applied one QALY decrement per RV infection. For example, despite that probably all hospitalised cases (except for nosocomial cases) would visit the GP before being hospitalised, only the QALY decrement for hospitalised cases were included in these cases.

### Costs

The analysis was performed from a societal perspective including both direct costs (health care and non-health care) and indirect costs of production losses, updated to 2010 (using the consumer price index from The Netherlands' Central Bureau of Statistics). Direct medical costs included in the analysis were drug costs (also including over-the-counter medication such as oral rehydration solutions and paracetamol), prescription fee for the pharmacist, cost of a GP consultation, and (nosocomial) hospitalisation costs (see Table 1 for specific costs per case). Additional costs of diapers and patient travel costs were included as direct non-medical costs [28]. Productivity losses included absence from work of the caregiver. Following Kemmeren *et al. *we assumed that the average sick leave duration of a caregiver corresponds to 23% of the average illness duration (for more details see Kemmeren *et al.*) and that approximately 13% of caregivers would be absent from work to care for a sick person [28]. Following the Dutch guidelines for heath economic evaluations we used a productivity elasticity of 0.8 to take into account compensation mechanisms for work losses.

In the absence of formal recommendations and reimbursement, the cost of the vaccine in the private market is between €125 and €150 for total vaccination. However, it is known that when a vaccine is included in the NIP and bulk quantities are bought by the government, large price reductions may occur during the tendering process. Therefore, we decided to calculate the maximum costs per vaccinated child considering a threshold of €20,000 and €50,000 per QALY gained based on the unofficial thresholds that are often applied to the Netherlands [29,30].

### Incremental cost-effectiveness calculations

The simulation model tracks cases of specific RVGE severities (see above), costs, savings and QALYs. Summing all cases, costs, savings and QALYs and consequently calculating the differences of the respective outcomes for evaluations with and without vaccination, rendered averted cases, net costs (costs minus savings) and QALYs gained. Dividing the net costs by either one of the health effects defined the ICER. Health effects (QALYs) and costs were discounted according to the Dutch guidelines for cost-effectiveness research by 1.5% and 4.0%, respectively [31].

### Sensitivity and scenario analyses

We performed univariate, multivariate and scenario analyses. As we did not use a fixed cost per vaccinated child in our analyses, we present the univariate and all scenario analyses using a total cost of €50, €75 and €100 per vaccinated child. To explore the impact of cost and utility parameters (other assumptions were varied in specific scenario analyses) relative to each other, a univariate sensitivity analysis was performed by varying the value of one parameter by 25%, while the other variables were kept constant at base-case values (often expected values of assumed distributions). This was represented in a Tornado diagram.

Several additional scenarios were considered. Recent epidemiological studies suggest the existence of herd protection benefits [32-38]. Also, many so-called dynamic models have been published which predict a herd effect in unvaccinated children [39,40]. Based on these studies, we explored the impact of inclusion of herd protection benefits for children (up to the age of 5 years) in the cohort. In this scenario we assumed herd protection for those not yet (fully) protected by the vaccine (either too young to be vaccinated or those who had not yet received the complete set of doses) and non-vaccinated children (5% of a birth cohort for the Dutch situation), assuming protection would be as effective as the vaccination would be after completing all doses.

Several previous cost-effectiveness analyses have incorporated QALY decrements for caregivers assuming their quality of life would be affected due to the fact that their children are ill [8,19]. When included, we assumed a decrement of 0.00184 or 0.0013 (= 0.00184 * 69%) for caregivers having a child requiring medical attention or for a case requiring no medical attention (child treated at home), respectively [8,25]. As noted above, in the base-case analysis we assumed that QALY decrement for cases treated at home would be 31% lower than cases requiring a GP visit based on the respective durations of illness. As this assumption might still be too conservative, we also explored the impact of applying a higher QALY loss for cases treated at home, which was similar to the QALY loss of cases treated by the GP. On the other extreme, we also explored a scenario in which no QALY losses were assumed for cases treated at home.

In the base-case analysis we used recent data provided by Mangen *et al. *for our epidemiological estimates [9]. An older study by Goossens *et al. *showed similar estimates with the exception of the incidence of RVGE-related GP visits which was more than twice as high [7]. We explored the impact of this higher GP incidence on the ICER.

We also investigated the impact of increasing the assumed mortality rate, based on earlier Dutch analyses which used much higher mortality rates. Mangen *et al.*, for example, used a mortality rate between 0.08% - 0.1% based on mortality rates observed in New York, USA [35]. We therefore investigated the impact of a mortality rate of 0.09% based on this study [35] and 0.055% which is in the mid-range of the latter estimate and our base-case estimate.

The impact of varying the productivity elasticity to labour time was also explored. Although the Dutch guidelines recommend a factor of 0.8 within the friction costing approach applied in the Netherlands, other international studies have included production losses as a straightforward multiplication of the wage and absence of work. On the other hand, it has been suggested that only 25% to 54% of conventionally included work loss should be taken into account when the time of absence is short [41]. Therefore, we investigated the impact of using elasticities of 100% and 25%. Finally, the impact of excluding indirect costs was explored as well as the impact of using other discount rates.

For probabilistic sensitivity analyses, parameters were generated using Monte Carlo sampling with outcome values being generated by running the model 5,000 times. Lognormal, normal and triangular distribution were used (Table 1), except for multinomial probabilities (in particular, the age-specific disease distribution) where Dirichlet distributions were assumed. Distributions for QALY decrements were calculated by applying the correction factor for age and severity (which were based on the UK study and was kept constant [26]) while varying the QALY decrement based on the Canadian study [25] assuming a normal distribution (mean of 0.0022; standard error [SE] of 0.00026).

## Results

### Cost-Effectiveness of RV Vaccination in the Base-Case Analysis

In the base-case analysis, the model estimates that in the birth cohort followed, 59,495 RVGE cases would occur resulting in 11,453 GP visits and 3,238 hospitalisations of which 421 are extensions of existing hospital stays due to nosocomial RV infection. With vaccination, 34,214 cases of RVGE would be averted corresponding to a total (discounted) QALY gain of 109 (see Table 2).

**Table 2:** Results from the base-case analysis

	Without vaccination	With vaccination^a^	Difference
Cases^b^	59,495	25,281	34,214

Treated at home	47,622	22,389	25,232

GP visits	11,453	2,786	8,667

Hospitalised (community acquired)	2,817	353	2,464

Hospitalised (nosocomial)	421	106	315

Deaths	0.65	0.16	0.48

Total QALYs lost^c^	173	64	109

Total direct costs^a ^(x1000)^c^	€ 7,470	€ 1,185	€ 6,282

Total indirect costs (x1000) ^c^	€ 2,193	€ 888	€ 1,305

^a ^Costs are excluding vaccination costs

^b ^Undiscounted

^c ^Discounted

**Figure 2:**
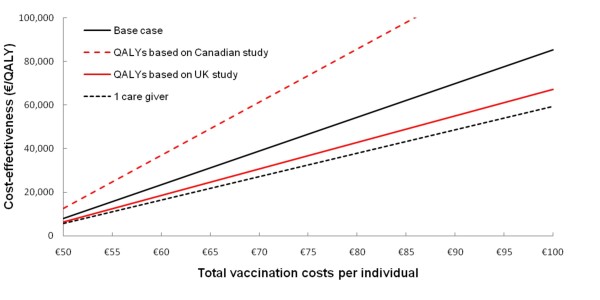
**Threshold analysis for various scenarios**. The solid black line shows the incremental cost-effectiveness ratio for the base-case analysis (no QALY losses for caregivers). The black dashed line show the ICER assuming QALY losses for 1 caregiver. The solid red line and the red dashed line shows the ICER when QALY losses were based on the UK or Canadian study, respectively [25,26]. Assuming a threshold of €20,000 or €50,000 per QALY specific threshold costs are €57.75 or €77.10 in the base case, €61.29 or €85.92 when the QALY losses were based on the UK study, €53.04 or €65.31 when the QALY losses were based on the Canadian study, and €63.39 or €91.23 assuming QALY losses for 1 caregiver.

In addition to the health gains, vaccination also prevents approximately €6.3 million of direct and €1.3 million of indirect costs. Applying thresholds for maximum willingness-to-pay of either €20,000 or €50,000 per QALY resulted in theoretical maximum total cost of €57.76 and €77.71 (see Figure 2).

### Sensitivity and scenario analyses

**Figure 3:**
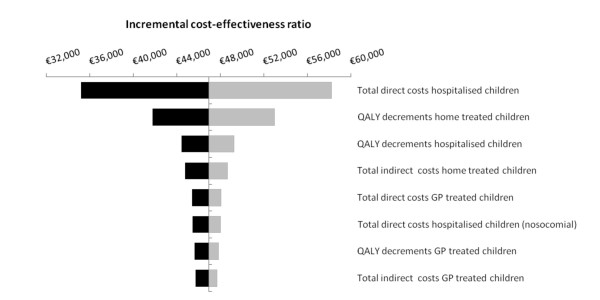
**Sensitivity analysis assumptions on the base-case cost-effectiveness ratio applying a total cost per vaccinated child of €75**. Parameters were varied by 25%. Black bars show the incremental cost-effectiveness ratio after a 25% decrease in the parameter, whereas grey bars show the incremental cost-effectiveness ratio after a 25% increase. Only parameters which changed the ICER by more than 1% are displayed. QALY: quality-adjusted life year; GP: general practitioner.

Figure 3 displays the results of the univariate sensitivity analysis (applying a total vaccine cost of €75). Apart from the total cost of vaccination (not included in the figure), the most influential parameters were the total direct costs associated with hospitalisation. Other parameters did not change the ICER by more than 15% when they were varied by 25%. The same parameters were also the main influential parameters for the same analysis performed using a higher (€100) or lower total vaccination cost (€50). However, when applying a lower total vaccine cost, the other costs included in the model become much more influential while an opposite effect was observed when a higher cost of vaccination was used (data not shown).

Figure 2 shows the impact on the ICER of varying the total cost per vaccinated child for different scenarios. Assuming that one caregiver would suffer QALY losses as well when their child was ill or applying QALY losses based on the UK study only [26], resulted in an increase of the maximum allowable theoretical cost of vaccination as compared to the base-case. On the other hand when utility losses were based solely on the Canadian study [25], the ICER was less favourable at a similar vaccination costs compared to the base-case analysis.

Table 3 shows that if vaccination led to indirect protective effects of unvaccinated individuals less than 5 years of age, the cost-effectiveness would greatly improve, and even become potentially cost-saving depending on the total cost of vaccination. Also, there was a large decrease in the ICER when a higher mortality rate was applied, when the incidence of GP visits was based on estimates by Goossens *et al. *[7], or QALY losses of children treated at home were assumed to be similar to those for children treated by the GP. In addition, the impact of changing the discount rates was considerable, for example, the ICER increased by 8-11% when an equal discount rate of 3.5% for costs and health effects was applied and decreased by 16-39% when neither costs nor effects were discounted. The scenario which resulted in the least favourable ICER was that when no QALY decrements were assumed in children treated at home.

**Table 3:** Scenario analyses

Scenario	ICER in €/QALY Total vaccination cost of €50	ICER in €/QALY Total vaccination cost of €75	ICER in €/QALY Total vaccination cost of €100
Base case	7,965	46,717	85,468

Inclusion of herd protection for children up to 5 years of age^a^	CS	28,383	58,441

GP incidence based on Goossens *et al. *[7]	498	35,855	71,211

			

DALYs based on Mangen *et al. *[9]	7,645	44,841	82,037

QALY decrements in children treated at home similar to cases visiting a GP	5,823	34,156	62,489

No QALY decrements in children treated at home	15,172	88,991	162,809

No waning	4,117	37,503	70,888

Mortality rate for hospitalised cases of 0.09%	4,627	27,140	49,653

Mortality rate for hospitalised cases of 0.055%	5,854	34,334	62,813

Productivity elasticity of 25%	16,184	54,936	93,688

No productivity elasticity	4,976	43,728	82,480

Excluding indirect costs (productivity losses)	19,921	58,672	97,424

No discounting	4,846	38,419	71,992

Equal discounting at 3.5%	8,587	51,892	95,197

CS = cost saving

^a ^Herd protection was assumed for those not yet (fully) protected by the vaccine (either too young to be vaccinated or those who had not yet received the complete set of doses) and non-vaccinated children (5% of a birth cohort for the Dutch situation), assuming protection would be as effective as the vaccination would be after completing all doses.

**Figure 4:**
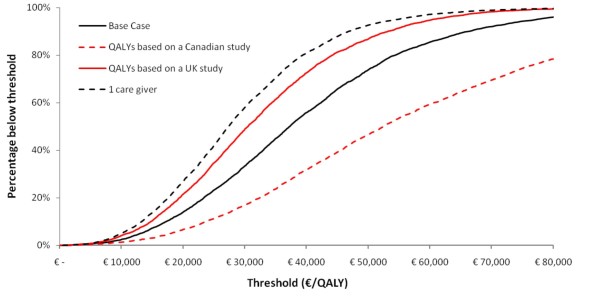
**Cost-effectiveness acceptability curves for base-case analysis and several other scenarios**.

Figure 4 shows the cost-effectiveness acceptability curves for the same scenarios, resulting from the threshold analysis. This figure shows that in the base-case analyses, 14% of the simulations resulted in an ICER of less than €20,000 per QALY. When a threshold of €50,000 per QALY was applied, 74% of the simulations resulted in acceptable ICERs. In all, but one of the remaining scenarios more than 70% of the simulations resulted in an ICER below €50,000 per QALY. When QALY estimates were based solely on the Canadian study [26], only 47% of the simulations resulted in an ICER below €50,000 per QALY.

## Discussion and conclusions

Our economic analysis indicates that inclusion of RV vaccination in the Dutch NIP could be considered cost-effective depending on the exact cost of the vaccine and the impact of RV on children's quality of life. Assumptions which have a major impact on the ICER and which are also associated with a relatively large degree of uncertainty are (i) the QALY losses associated with RVGE, particularly in children treated at home and in caregivers, (ii) inclusion of potential herd protection, and (iii) the mortality rate in hospitalised RVGE cases. Along with assumed differences in underreporting, these aspects also explain the variation in the outcomes of the cost-effectiveness analyses performed by other research groups.

### Strengths and weaknesses

In the base-case analysis, we chose not to include indirect protective effects for unvaccinated individuals within (approximately 5% in the Netherlands) and outside the vaccinated cohort. Recent epidemiological studies do, however, provide some evidence for the existence of such herd protection benefits [32-38]. Additionally, several so-called dynamic models have been published which also predict an indirect protective effect in unvaccinated children [39,40]. However, further evidence is required before definite interpretations can be made. Therefore, we did not include these indirect protective effects in our base-case analysis as we did not want to present a too optimistic picture on the cost-effectiveness, which has been the case previously with pneumococcal vaccination [29,30]. Yet, conservative inclusion of limited potential herd protection effects in children (those aged less than 5 years of age) could improve cost-effectiveness considerably.

QALY losses of caregivers were not included in the base-case analysis. The impacts on the quality of life of caregivers are generally not included in Dutch cost-effectiveness evaluations, and including them here would have made a comparison with other interventions difficult (see below). Nevertheless, the Dutch pharmacoeconomic guidelines indicate that from a societal perspective, all costs and benefits should be considered, irrespective of who pays or loses, and who benefits [42]. This would certainly provide an argument in favour of including all QALY impacts, such as those on caregivers. If we did incorporate QALY losses of caregivers, the ICER decreased considerably. It is not unlikely that a child suffering from RV has a similar QALY impact on both parents, and including two caregivers in the analysis could even be advocated. However, given that not all families consist of two caregivers and our approach is to remain conservative, this analysis was not pursued here.

Besides the assumed QALY decrements for caregivers, especially the assumed QALY losses for children treated at home had a major impact on the ICER. We based our QALY decrement for children on two published studies performed in the UK and Canada - the only ones currently available in the literature [25,26]. In the UK study, the utility of infants suffering from an RV infection was determined by health care providers, while in the Canadian study the utility decrements were estimated by the caregivers of children visiting a GP or paediatrician because of RVGE. Which of these estimates is more appropriate is not easy to determine. The Canadian study based their estimates on parents, which might be more suitable when performing a cost-effectiveness study from a societal perspective than estimates from GPs. On the other hand, the UK study provided age- and disease-severity-specific estimates, which might be more appropriate than one overall QALY decrement. We therefore chose to base our estimates on combining both studies (as described in the Methods). We do, however, note that combining the data from these different studies come with limitations. For example, the utility estimates and the duration of illness, which were used to estimate the QALY loss per case came from different foreign countries where one would ideally wanted to have those from one and the same study. Given differences in health-care systems and treatment patterns, combining information on duration and utilities from different countries may provide non-optimal estimations.

In contrast to previous studies, which used 50% of the QALY loss of cases attending primary care for cases not seeking medical care having, we assumed that the QALY loss in cases that would be treated at home would be 31% lower than for cases requiring a GP visit, based on illness durations. However, we feel that the former approach is likely to underestimate the QALY loss in these cases as only the most severe RVGE cases are expected to visit a primary care facility in the Netherlands (see also below). Although we are aware of the limitation of our approach, we do feel that this is the best approach. To anticipate on the uncertainty, we performed extensive sensitivity analyses on the QALY losses per case not seeking medical care.

We based the mortality rate (0.02% in hospitalised cases) on a study performed in England and Wales [18]. Applying this rate in our model resulted in 0.65 deaths for all children less than 5 years of age (assuming a birth cohort of 180,000 infants each year). Increasing the mortality rates to 0.055% or 0.09% decreased the ICER to 27% and 42%, respectively. When we applied higher mortality rates, changes in the discount rate for health effects had a larger impact on the ICER, since the life years were obtained over a long time period.

The GP incidence used in our study is lower compared to those observed and used in other countries. As previously argued by Mangen *et al. *this is likely to be related to the fact that in the Netherlands it is common practice to advise persons with GE to consult a GP only if symptoms remain for a longer period, or if the patient's health state gets worse [9]. Furthermore, in the Netherlands it is not required to obtain a medical certificates from a GP to prove sickness or having a sick child at home. In other European countries like Germany, France and Spain such a certificate is required within 1-3 days off work in order to take care of a sick person, consequently GP's in these countries will be consulted more often [9].

In contrast to previous studies, we based our efficacy estimates on specific European vaccine efficacy data wherever possible [21,23,24]. Using these data instead of the general efficacy data (which were based on 11 countries throughout the world) probably gives more reliable estimates. We used efficacy estimates based on the latest available data for RotaTeq^®^. These data show that the efficacy estimates after the second dose of RotaTeq^® ^are much more similar to the efficacy of Rotarix^® ^after the second dose than previously assumed [11]. Also, remaining differences between both vaccines' efficacy estimates are based on clinical trials performed in different regions of the world and case definitions for disease were different between clinical trials performed with Rotateq^® ^and Rotarix^® ^[19]. Strictly considered, our analysis - building on Rotateq^® ^clinical trials - is an economic evaluation for that specific vaccine, yet we expect the results for a Rotarix^®^-specific analysis to be highly similar given the similarities between both vaccines.

### Comparison with other studies

Our calculated cost-effectiveness ratio for RV vaccination is in between the estimates of previous Dutch studies [7-10]. This is due to a combination of factors: (i) we used a lower total cost per vaccinee, (ii) we used higher QALY decrements in our study than in three of the four previous studies [8-10], (iii) we used more realistic disease incidence data (including mortality rates) compared to all previous studies, and finally (iv) we estimated efficacy based on the most suitable data.

On the one hand, our results indicate that RV vaccination is probably more cost-effective than the current Dutch pneumococcal vaccination programme with the seven valent pneumococcal vaccine [30]. On the other hand, our cost-effectiveness results show that RV vaccination is likely to be more expensive per QALY gained than other routine vaccination programs recently implemented such as HPV [43] (€30,000 per QALY). It is as yet unclear how RV vaccination compares to other vaccination programs not yet implemented in the Netherlands, such as for varicella [44]. Yet, the cost-effectiveness crucially depends on the exact vaccination costs of the RV vaccine if included within the Dutch NIP.

### Implications and future research

Increasingly crowded infant vaccination schedules and restrained national budgets highlight the importance of cost-effectiveness analyses in the decision-making process on which vaccines should be included in national immunisation programmes. We show that RV vaccination in the Netherlands can be considered cost-effective depending on the total cost per vaccinated child. We also describe the main drivers for cost-effectiveness outcomes. In order to make an accurate appraisal of the RV vaccine and other currently available - but not yet introduced - vaccines as well as upcoming vaccines such as respiratory syncytial virus (RSV) vaccines, more accurate data regarding the main uncertain cost-effectiveness drivers are necessary.

Future research should, therefore, focus in particular on the number of deaths due to RV infections in the Netherlands as accurate data for the Netherlands and most other European countries are currently lacking. In addition, the relatively old cohort studies conducted at the population and GP level [12-15] should ideally be updated, in combination with a cohort study conducted at hospital level. Furthermore, more research is needed on the quality of life of infected children. Consensus should be obtained regarding the question whether or not to incorporate the effect of childhood disease on the quality of life of caregivers [19]. Finally, as potential herd effects have a large impact on the cost-effectiveness, continued surveillance and additional epidemiological studies in those countries in which an RV vaccination schedule has already been introduced should provide more insights into the epidemiology of RV over time, including such potential indirect effects.

## List of abbreviations

RV: rotavirus; NIP: national Immunization Program; RVGE: rotavirus gastroenteritis; GP: general practitioner; ED: emergency department; CI: confidence interval; QALY: quality-adjusted life year; DALYs: disability-adjusted life year; ICER: incremental cost-effectiveness ratio; SE: standard error; RSV: respiratory syncytial virus

## Competing interests

This research was supported by an unrestricted grant from SPMSD. Consensus meetings were logistically facilitated by SPMSD, formal invitations came from the University of Groningen, reimbursement of travel and time costs was settled within the framework of another grant by SPMSD to the University of Groningen specifically designed for this. Lotte Westerink (MSc-student in Pharmacy at the University of Groningen) supported the preparation of the meetings during an internship at SPMSD (Lyon, France) within the framework of the MSc Course on "Policy & Management" at the Faculty of Mathematics and Natural Sciences of the University of Groningen.

## Authors' contributions

MJP and EH designed the study. MHR and MJP designed the computer model and carried out the computer simulations and analysis. Data analyses were performed by MJJM and MHR under supervision of MJP, EH. MHR, MJJM and MJP drafted the manuscript. CG and JCW commented on the drafts and advised in various stages of the research. All authors commented on, contributed to and approved the final version of the manuscript.

## Pre-publication history

The pre-publication history for this paper can be accessed here:

http://www.biomedcentral.com/1471-2458/11/462/prepub
